# Synthesis and crystal structure of Sr_2_Cu(OH)_4_[B(OH)_4_]_2_

**DOI:** 10.1107/S2056989025011491

**Published:** 2026-01-01

**Authors:** Hibiki Kunisawa, Jun-ichi Yamaura, Toshihiro Nomura

**Affiliations:** a Department of Physics, Shizuoka University, Shizuoka 422-8529, Japan; b Institute for Solid State Physics, University of Tokyo, Kashiwa, Chiba 277-8581, Japan; Vienna University of Technology, Austria

**Keywords:** crystal structure, inorganic, hy­droxy­borate

## Abstract

Single crystals of distrontium copper(II) tetra­hydroxide bis­(tetra­hydroxidoborate), Sr_2_Cu(OH)_4_[B(OH)_4_]_2_, were obtained by an ammonia evaporation method at room temperature. The compound crystallizes in the triclinic system, space group *P*1, and is isotypic with the calcium analogue henmilite, Ca_2_Cu(OH)_4_[B(OH)_4_]_2_. The {Cu(OH)_4_} units form a deformed square lattice in the *ac* plane, giving rise to a quasi-two-dimensional arrangement of Cu^II^ ions. An intricate network of O—H⋯O hydrogen bonds of medium strengths with the [B(OH)_4_] units as the primary donor groups consolidate the framework structure.

## Abstract 2

The isotypic strontium analogue of the mineral henmilite, Sr_2_Cu(OH)_4_[B(OH)_4_]_2_, displays a quasi two-dimensional spin system with Cu^II^ ions.

## 1. Chemical context

Hydroxidoborates containing Cu^II^ ions are of inter­est owing to their structural diversity and potential magnetic frustration. Among them is the mineral henmilite, Ca_2_Cu(OH)_4_[B(OH)_4_]_2_ (space group *P*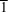) (Nakai *et al.*, 1986 ▸; Nakai, 1986 ▸; Petrov, 2016 ▸), that exhibits a layered framework where {Cu(OH)_4_} units form a quasi two-dimensional spin system (Yamamoto *et al.*, 2021 ▸; Hayashi *et al.*, 2023 ▸). Some basic properties of henmilite are reported elsewhere (Kusachi, 1992 ▸; Frost & Xi, 2013 ▸). Yamamoto *et al.* proposed the doubled unit cell containing two Cu^II^ ions compared to the original report by Nakai (**a′** = 2**a**, **b′** = **b**, **c′** = **c**), suggesting an alternating Cu⋯Cu distance along the *a* axis. Thus, the exchange parameters are also alternatively modulated, and the system can be regarded as a coupled two-leg spin ladder (Yamamoto *et al.*, 2021 ▸). The anti­ferromagnetic ordering temperature is reported to be approximately 0.2 K at zero magnetic fields.

In the present study, we have synthesized the strontium analogue, Sr_2_Cu(OH)_4_[B(OH)_4_]_2_, by an ammonia evaporation method at room temperature and report here its crystal structure.

## 2. Structural commentary

**Figure 1:**
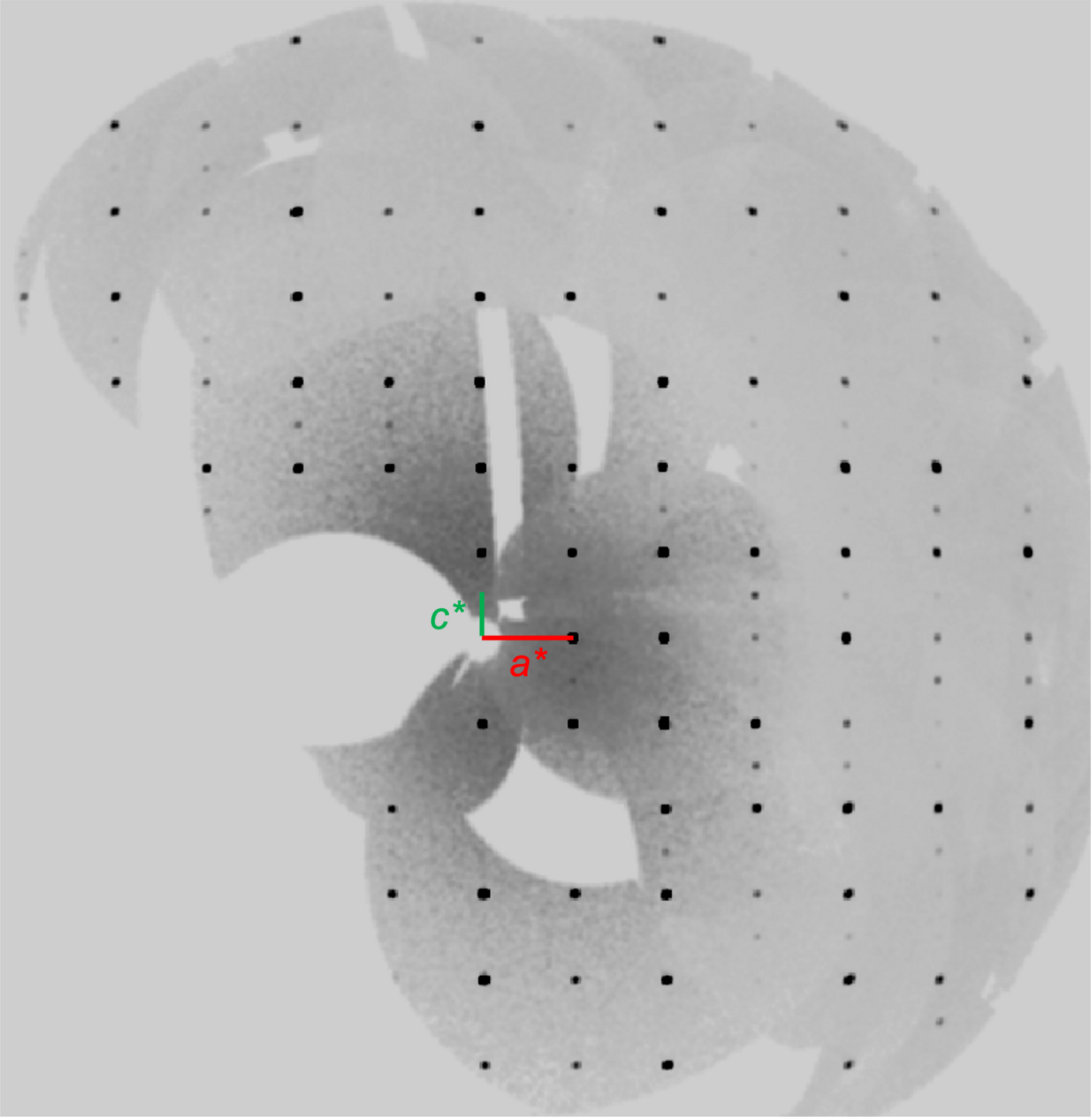
Reconstructed precession image of the (*h*0*l*) plane, showing weak superstructure reflections at odd *l* positions (corresponding to half-integer *l* in the halved cell), consistent with a doubling of the unit cell along the *c* direction.

Sr_2_Cu(OH)_4_[B(OH)_4_]_2_ crystallizes in space group *P*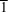 (Figs. 1 ▸ and 2 ▸) and is isotypic with henmilite. The mineral was described using a non-standard settings both for the basis cell (Nakai *et al.*, 1986 ▸; Nakai, 1986 ▸) and for the doubled unit cell (Yamamoto *et al.*, 2021 ▸). The current description of the Sr isotype uses the standard setting with the transformation of **a′′** = **c′**, **b′′** = **b′**, **c′′** = **a′**, where the single primed parameters relate to the doubled unit cell reported by Yamamoto *et al.* (2021 ▸). We note that the crystal structure of the title compound is also doubled as clearly demonstrated by the superlattice reflections shown in Fig. 1 ▸.

**Figure 2:**
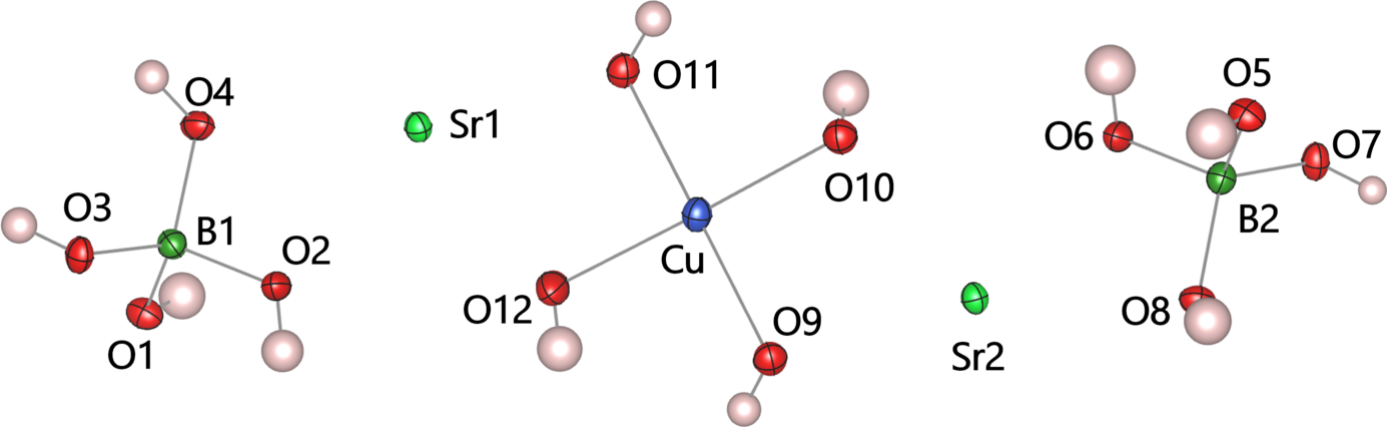
A view of the asymmetric unit of Sr_2_Cu(OH)_4_[B(OH)_4_]_2_. Displacement ellipsoids are drawn at the 50% probability level.

**Figure 3:**
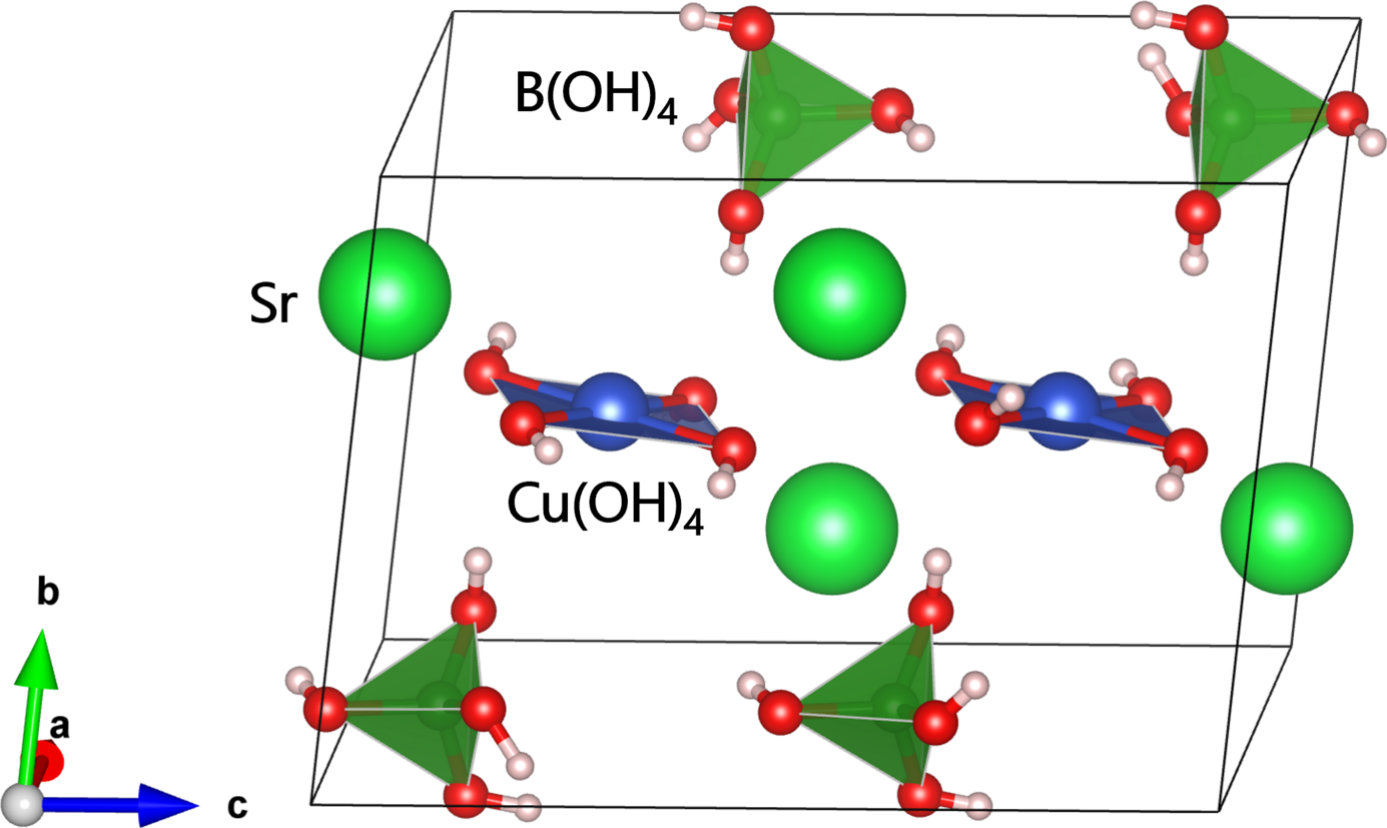
Crystal structure of Sr_2_Cu(OH)_4_[B(OH)_4_]_2_.

The crystal structure is composed of square-planar {Cu(OH)_4_} units, {Sr(OH)_8_} polyhedra, and tetra­hedral [B(OH)_4_] units (Fig. 2 ▸). The {Sr(OH)_8_} polyhedra centred by the Sr1 and Sr2 sites are close to square anti­prisms, but are notably distorted in the triclinic lattice. The edge-sharing {Sr(OH)_8_} units form chains extending parallel to [110], which are inter­connected by {Cu(OH)_4_} and [B(OH)_4_] units into a framework structure (Fig. 3 ▸). As a result, Cu^II^ ions form a quasi two-dimensional system in the *ac* plane arranged in a deformed square lattice. The nearest-neighbour distance between Cu^II^ ions is 5.84880 (11) Å along the *a* axis. Along the *c* axis, the Cu⋯Cu distances alternate between 5.8959 (5) Å and 5.9681 (5) Å.

## 3. Supra­molecular features

**Figure 4:**
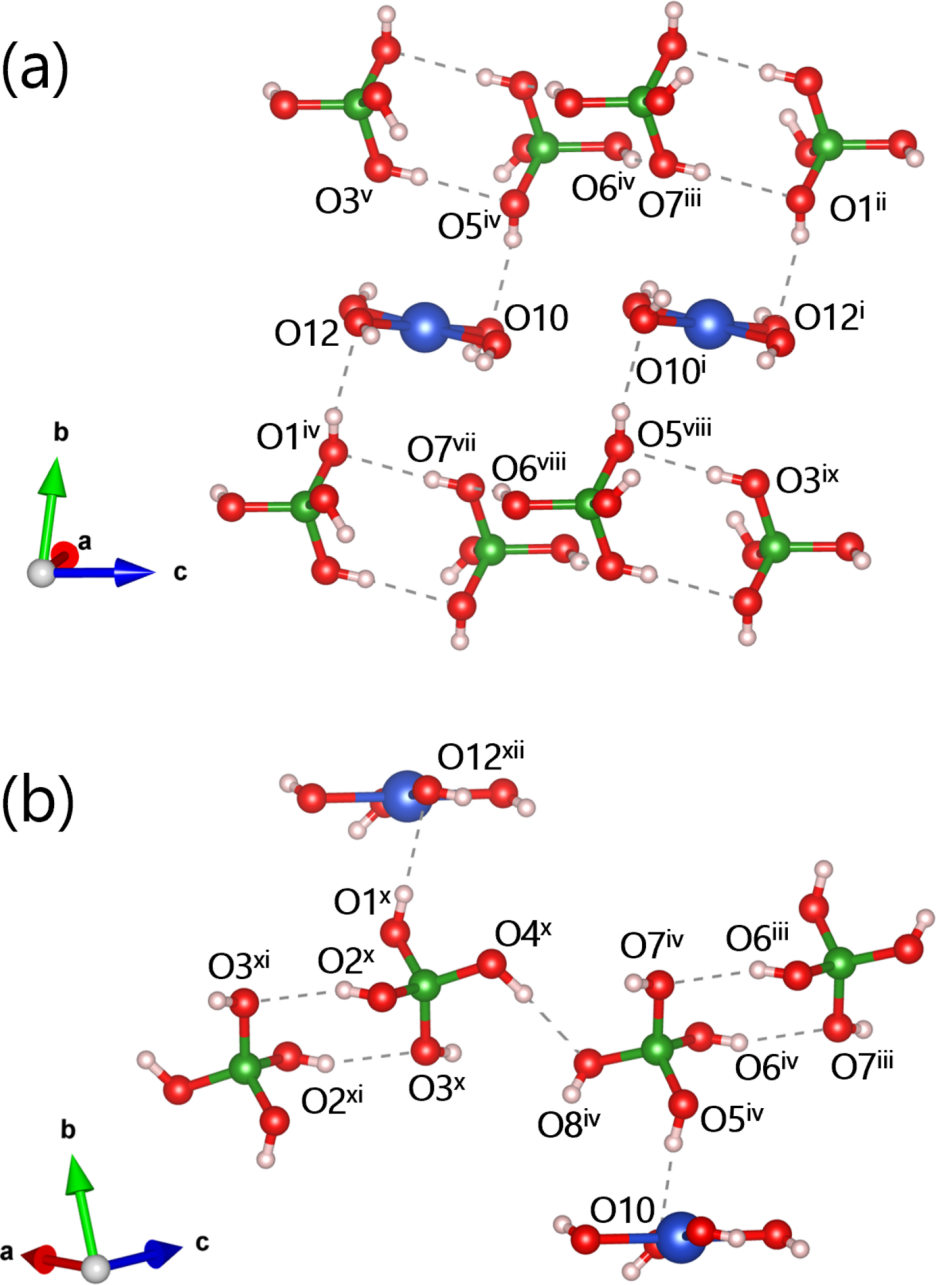
Hydrogen-bonding network in Sr_2_Cu(OH)_4_[B(OH)_4_]_2_ in different views (*a*) and (*b*). Sr^II^ ions are omitted for clarity. [Symmetry codes: (i) −*x* + 1, −*y* + 1, −*z* + 1; (ii) *x* + 1, *y*, *z* + 1; (iii) *x* − 1, *y* + 1, *z*; (iv) −*x* + 2, −*y* + 1, −*z* + 1; (v) −*x* + 1, −*y* + 2, −*z*; (vi) −*x*, −*y* + 1, −*z*; (vii) −*x* + 2, −*y*, −*z* + 1; (viii) *x* − 1, *y*, *z*; (ix) *x* + 1, *y* − 1, *z* + 1; (x) −*x* + 1, −*y* + 2, −*z*; (xi) *x* + 2, *y*, *z*; (xii) *x* + 1, *y* + 1, *z*.]

In the three-dimensional hydrogen-bonding network of Sr_2_Cu(OH)_4_[B(OH)_4_]_2_, tetra­hedral [B(OH)_4_] units play a central role (Table 1 ▸, Fig. 4 ▸). The [B1(OH)_4_] tetra­hedron donates via atoms H1–H4 and accepts via O1 and O3 atoms, while the [B2(OH)_4_] tetra­hedron donates via atoms H5–H7 and accepts via O5, O7, and O8 with surrounding [B(OH)_4_] tetra­hedra and {Cu(OH)_4_} plaquettes. The hydrogen-bonding distances of 2.35 Å at O8—H8⋯O12 and 2.39 Å O12—H12⋯O10 are relatively long and omitted in Fig. 4 ▸. The hydrogen-bonding network is topologically identical in henmilite and the title compound.

We note that the hydrogen atoms H9, H10, and H11 of the {Cu(OH)_4_} unit are not involved in notable hydrogen-bonding inter­actions with the surrounding [B(OH)_4_] and {Cu(OH)_4_} units. A similar situation is observed in henmilite, where the hydrogen bonds donated by the{Cu(OH)_4_} unit are relatively long (two of them exceed 2.4 Å). In the title compound, the larger unit-cell volume (by 9.4%) results in increased inter­molecular separations, and consequently such hydrogen bonds are absent.

## 4. Database survey

**Figure 5:**
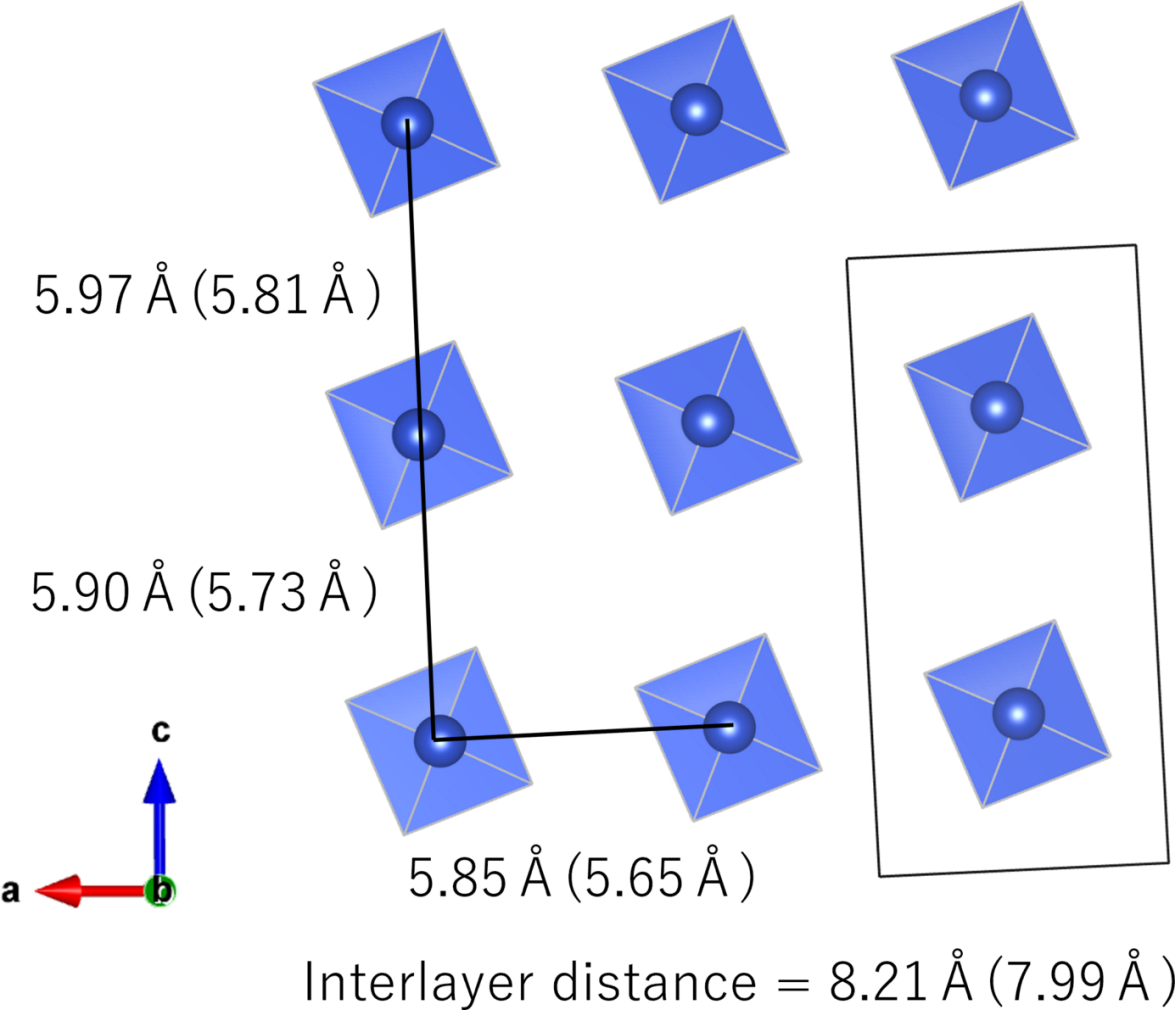
Cu sublattice in Sr_2_Cu(OH)_4_[B(OH)_4_]_2_ with Cu⋯Cu distances. Values in parentheses are the corresponding distances in Ca_2_Cu(OH)_4_[B(OH)_4_]_2_ (Yamamoto *et al.*, 2021 ▸). The crystallographic axes and unit cell are drawn for Sr_2_Cu(OH)_4_[B(OH)_4_]_2_.

According to the Inorganic Crystal Structure Database (ICSD (version 2025-1), FIZ Karlsruhe; Zagorac *et al.*, 2019 ▸), no hydroxidoborates containing Cu^II^ and alkaline-earth metal ions have been reported, except for henmilite. Based on the layered structure, Sr_2_Cu(OH)_4_[B(OH)_4_]_2_ is also expected to show features of a low-dimensional spin system. Due to the larger ionic radius of Sr^II^, Cu⋯Cu distances in Sr_2_Cu(OH)_4_[B(OH)_4_]_2_ are longer than those in henmilite as summarized in Fig. 5 ▸. This indicates that the exchange inter­action in Sr_2_Cu(OH)_4_[B(OH)_4_]_2_ is weaker than in henmilite, and hence, the ordering temperature is expected to be even lower than 0.2 K.

## 5. Synthesis and crystallization

Single crystals were grown by slow ammonia evaporation at room temperature. A mixture of CuCO_3_·Cu(OH)_2_ (0.1 g), H_3_BO_3_ (0.2 g), and Sr(OH)_2_·8H_2_O (1.6 g) was dissolved in 20 ml of 5%_wt_ aqueous ammonia. All reagents were purchased from FUJIFILM Wako and used as received. The beaker was placed in a sealed container at room temperature together with 6%_wt_ nitric acid to control the evaporation rate. After 1–2 weeks, violet–blue rhombic crystals were obtained. We picked a 30 × 30 × 10 µm^3^ single crystal and performed the XRD at room temperature. We note that a powder sample of henmilite can also be obtained in a similar method, starting from Ca(OH)_2_ instead of Sr(OH)_2_·8H_2_O.

## 6. Refinement

The crystallographic data, data collection and structure refinement details are summarized in Table 2 ▸. Hydrogen atoms were located from difference syntheses. Refinement trials were carried out with both *AFIX* and *DFIX* restraints (Sheldrick, 2015 ▸) on hydrogen atoms. The *AFIX*-based model converged with *R*_1_ = 0.0203, but yielded unrealistic O—H geometries. Therefore, all O—H distances were restrained to 0.82 (5) Å using *DFIX*, giving a final *R*_1_ = 0.0178.

## Supplementary Material

Crystal structure: contains datablock(s) I. DOI: 10.1107/S2056989025011491/wm5783sup1.cif

Structure factors: contains datablock(s) I. DOI: 10.1107/S2056989025011491/wm5783Isup3.hkl

CCDC reference: 2517776

Additional supporting information:  crystallographic information; 3D view; checkCIF report

## Figures and Tables

**Table 1:** Hydrogen-bond geometry (Å, °)

*D*—H⋯*A*	*D*—H	H⋯*A*	*D*⋯*A*	*D*—H⋯*A*
O1—H1⋯O12^i^	0.81 (3)	2.10 (3)	2.886 (2)	164 (3)
O2—H2⋯O3^ii^	0.77 (3)	2.07 (3)	2.8060 (18)	162 (3)
O3—H3⋯O5^iii^	0.82 (2)	2.07 (2)	2.8563 (19)	163 (2)
O4—H4⋯O8^iv^	0.89 (2)	1.93 (2)	2.8125 (18)	176 (2)
O5—H5⋯O10^v^	0.81 (3)	2.01 (3)	2.7961 (19)	163 (3)
O6—H6⋯O7^vi^	0.79 (3)	2.05 (3)	2.8129 (18)	162 (3)
O7—H7⋯O1^vii^	0.78 (2)	2.10 (2)	2.8592 (19)	166 (2)
O8—H8⋯O12^viii^	0.74 (3)	2.35 (3)	3.073 (2)	166 (3)
O12—H12⋯O10^ix^	0.72 (3)	2.39 (3)	3.1076 (19)	171 (3)

Symmetry codes: (i) 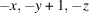; (ii) 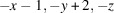; (iii) 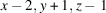; (iv) 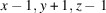; (v) 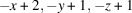; (vi) 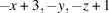; (vii) 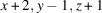; (viii) 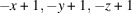; (ix) 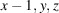.

**Table 2:** Experimental details

Crystal data	
Chemical formula	Sr_2_Cu(OH)_4_[B(OH)_4_]_2_
*M*_r_	464.51
Crystal system, space group	Triclinic, *P* 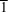
Temperature (K)	293
*a*, *b*, *c* (Å)	5.8488 (1), 8.2051 (2), 11.8619 (3)
α, β, γ (°)	83.789 (2), 87.614 (2), 70.499 (2)
*V* (Å^3^)	533.44 (2)
*Z*	2
Radiation type	Cu *K*α
μ (mm^−1^)	15.83
Crystal size (mm)	0.03 × 0.03 × 0.01

Data collection	
Diffractometer	Four-circle diffractometer
Absorption correction	Multi-scan (*CrysAlis PRO*; Rigaku OD, 2023 ▸)
*T*_min_, *T*_max_	0.763, 1.000
No. of measured, independent and observed [*I* > 2σ(*I*)] reflections	6151, 2224, 1927
*R*_int_	0.014
(sin θ/λ)_max_ (Å^−1^)	0.635

Refinement	
*R*[*F*^2^ > 2σ(*F*^2^)], *wR*(*F*^2^), *S*	0.018, 0.055, 1.09
No. of reflections	2224
No. of parameters	203
No. of restraints	12
H-atom treatment	All H-atom parameters refined
Δρ_max_, Δρ_min_ (e Å^−3^)	0.41, −0.60

Computer programs: *CrysAlis PRO* (Rigaku OD, 2023 ▸), *OLEX2.solve* (Bourhis *et al.*, 2015 ▸), *SHELXL* (Sheldrick, 2015 ▸), *VESTA 3* (Momma & Izumi, 2011 ▸) and *publCIF* (Westrip, 2010 ▸).

## supplementary crystallographic information

### Strontium copper(II) tetrahydroxide bis(tetrahydroxyborate) Crystal data

**Table:**

Sr_2_Cu(OH)_4_[B(OH)_4_]_2_	*Z* = 2
*M**_r_* = 464.51	*F*(000) = 446
Triclinic, *P*1	*D*_x_ = 2.892 Mg m^−^^3^
*a* = 5.8488 (1) Å	Cu *K*α radiation, λ = 1.54184 Å
*b* = 8.2051 (2) Å	Cell parameters from 3611 reflections
*c* = 11.8619 (3) Å	θ = 5.7–77.7°
α = 83.789 (2)°	µ = 15.83 mm^−^^1^
β = 87.614 (2)°	*T* = 293 K
γ = 70.499 (2)°	Plate, translucent, blue-violet
*V* = 533.44 (2) Å^3^	0.03 × 0.03 × 0.01 mm

### Strontium copper(II) tetrahydroxide bis(tetrahydroxyborate) Data collection

**Table:**

Four-circle diffractometer	*R*_int_ = 0.014
Radiation source: Rotating-anode X-ray tube	θ_max_ = 78.1°, θ_min_ = 3.8°
ω scans	*h* = −6→7
Absorption correction: multi-scan (CrysAlisPro; Rigaku OD, 2023)	*k* = −10→10
*T*_min_ = 0.763, *T*_max_ = 1.000	*l* = −13→14
6151 measured reflections	Standard reflections: not measured; every not measured reflections
2224 independent reflections	intensity decay: not measured
1927 reflections with *I* > 2σ(*I*)	

### Strontium copper(II) tetrahydroxide bis(tetrahydroxyborate) Refinement

**Table:**

Refinement on *F*^2^	Hydrogen site location: difference Fourier map
Least-squares matrix: full	All H-atom parameters refined
*R*[*F*^2^ > 2σ(*F*^2^)] = 0.018	*w* = 1/[σ^2^(*F*_o_^2^) + (0.036*P*)^2^ + 0.0131*P*] where *P* = (*F*_o_^2^ + 2*F*_c_^2^)/3
*wR*(*F*^2^) = 0.055	(Δ/σ)_max_ = 0.001
*S* = 1.09	Δρ_max_ = 0.41 e Å^−^^3^
2224 reflections	Δρ_min_ = −0.60 e Å^−^^3^
203 parameters	Extinction correction: SHELXL-2019/2 (Sheldrick 2015), Fc^*^=kFc[1+0.001xFc^2^λ^3^/sin(2θ)]^-1/4^
12 restraints	Extinction coefficient: 0.00104 (13)

### Strontium copper(II) tetrahydroxide bis(tetrahydroxyborate) Special details

**Table:**

Geometry. All esds (except the esd in the dihedral angle between two l.s. planes) are estimated using the full covariance matrix. The cell esds are taken into account individually in the estimation of esds in distances, angles and torsion angles; correlations between esds in cell parameters are only used when they are defined by crystal symmetry. An approximate (isotropic) treatment of cell esds is used for estimating esds involving l.s. planes.

### Strontium copper(II) tetrahydroxide bis(tetrahydroxyborate) Fractional atomic coordinates and isotropic or equivalent isotropic displacement parameters (Å^2^)

**Table:**

	*x*	*y*	*z*	*U*_iso_*/*U*_eq_	
Sr1	0.26270 (2)	0.74355 (2)	0.00346 (2)	0.01241 (9)	
Sr2	0.72436 (3)	0.25409 (2)	0.49668 (2)	0.01266 (9)	
Cu	0.49102 (3)	0.49916 (3)	0.25177 (2)	0.01478 (10)	
B1	−0.2578 (4)	0.9087 (3)	−0.11486 (17)	0.0133 (4)	
B2	1.2500 (4)	0.0957 (3)	0.61569 (17)	0.0138 (4)	
O1	−0.4028 (2)	0.79592 (18)	−0.13154 (11)	0.0159 (3)	
O2	−0.1918 (2)	0.89861 (17)	0.00634 (11)	0.0157 (3)	
O3	−0.4046 (2)	1.09031 (16)	−0.14559 (11)	0.0153 (3)	
O4	−0.0262 (2)	0.85237 (17)	−0.17663 (11)	0.0166 (3)	
O5	1.3820 (2)	0.21430 (18)	0.63596 (12)	0.0168 (3)	
O6	1.1846 (2)	0.10388 (17)	0.49505 (11)	0.0161 (3)	
O7	1.4086 (2)	−0.08390 (16)	0.64487 (12)	0.0160 (3)	
O8	1.0151 (2)	0.13438 (17)	0.67660 (11)	0.0166 (3)	
O9	0.3483 (2)	0.47831 (17)	0.40103 (11)	0.0166 (3)	
O10	0.8087 (2)	0.43586 (17)	0.32424 (12)	0.0177 (3)	
O11	0.6323 (2)	0.52314 (16)	0.10290 (11)	0.0161 (3)	
O12	0.1793 (2)	0.56215 (19)	0.17586 (13)	0.0220 (3)	
H1	−0.320 (5)	0.695 (4)	−0.135 (3)	0.032 (7)*	
H2	−0.290 (5)	0.879 (4)	0.045 (2)	0.026 (7)*	
H3	−0.435 (4)	1.117 (3)	−0.213 (2)	0.019 (6)*	
H4	−0.009 (3)	0.938 (3)	−0.225 (2)	0.017 (5)*	
H5	1.300 (5)	0.315 (4)	0.642 (3)	0.038 (8)*	
H6	1.284 (5)	0.121 (4)	0.453 (3)	0.037 (8)*	
H7	1.436 (4)	−0.109 (3)	0.709 (2)	0.012 (6)*	
H8	0.991 (4)	0.201 (4)	0.717 (2)	0.033 (7)*	
H9	0.232 (4)	0.453 (4)	0.399 (2)	0.017 (6)*	
H10	0.891 (4)	0.372 (4)	0.286 (2)	0.031 (7)*	
H11	0.756 (4)	0.548 (4)	0.108 (2)	0.019 (7)*	
H12	0.089 (4)	0.543 (4)	0.213 (2)	0.030 (7)*	

### Strontium copper(II) tetrahydroxide bis(tetrahydroxyborate) Atomic displacement parameters (Å^2^)

**Table:**

	*U*^11^	*U*^22^	*U*^33^	*U*^12^	*U*^13^	*U*^23^
Sr1	0.01143 (11)	0.01167 (12)	0.01307 (12)	−0.00243 (7)	−0.00086 (7)	−0.00093 (7)
Sr2	0.01190 (11)	0.01148 (12)	0.01352 (12)	−0.00246 (7)	−0.00113 (7)	−0.00087 (7)
Cu	0.01638 (18)	0.01437 (17)	0.01296 (18)	−0.00402 (13)	−0.00138 (13)	−0.00159 (12)
B1	0.0125 (9)	0.0130 (9)	0.0143 (10)	−0.0042 (7)	−0.0006 (7)	−0.0008 (7)
B2	0.0146 (9)	0.0134 (9)	0.0130 (10)	−0.0045 (7)	−0.0009 (7)	−0.0004 (7)
O1	0.0142 (6)	0.0138 (6)	0.0202 (7)	−0.0051 (5)	0.0012 (4)	−0.0032 (5)
O2	0.0122 (5)	0.0221 (6)	0.0122 (6)	−0.0053 (5)	0.0003 (5)	−0.0005 (5)
O3	0.0167 (6)	0.0118 (6)	0.0149 (7)	−0.0013 (5)	−0.0025 (5)	−0.0002 (5)
O4	0.0139 (5)	0.0161 (6)	0.0172 (6)	−0.0025 (4)	0.0035 (4)	0.0005 (5)
O5	0.0160 (6)	0.0143 (6)	0.0210 (7)	−0.0061 (5)	0.0005 (5)	−0.0028 (5)
O6	0.0132 (6)	0.0221 (6)	0.0127 (6)	−0.0058 (5)	−0.0004 (4)	−0.0002 (5)
O7	0.0172 (6)	0.0132 (6)	0.0144 (7)	−0.0007 (5)	−0.0033 (5)	−0.0007 (5)
O8	0.0148 (6)	0.0180 (6)	0.0167 (7)	−0.0048 (5)	0.0033 (5)	−0.0039 (5)
O9	0.0167 (6)	0.0173 (6)	0.0149 (7)	−0.0041 (5)	0.0004 (5)	−0.0031 (5)
O10	0.0171 (6)	0.0185 (6)	0.0170 (7)	−0.0051 (5)	−0.0007 (5)	−0.0019 (5)
O11	0.0169 (6)	0.0157 (6)	0.0145 (7)	−0.0035 (5)	−0.0006 (5)	−0.0026 (5)
O12	0.0183 (6)	0.0297 (7)	0.0192 (7)	−0.0114 (6)	−0.0034 (5)	0.0044 (6)

### Strontium copper(II) tetrahydroxide bis(tetrahydroxyborate) Geometric parameters (Å, º)

**Table:**

Sr1—Cu	3.4266 (3)	Cu—O9	1.9410 (13)
Sr1—Cu^i^	3.7726 (3)	Cu—O10	1.9630 (13)
Sr1—B1^ii^	3.263 (2)	Cu—O11	1.9369 (13)
Sr1—B1	3.204 (2)	Cu—O12	1.9516 (13)
Sr1—O1^iii^	2.5936 (13)	B1—O1	1.481 (2)
Sr1—O2	2.5358 (12)	B1—O2	1.491 (2)
Sr1—O2^ii^	2.8149 (13)	B1—O3	1.465 (2)
Sr1—O3^ii^	2.5996 (13)	B1—O4	1.469 (2)
Sr1—O4	2.6640 (13)	B2—O5	1.472 (2)
Sr1—O11^i^	2.5285 (12)	B2—O6	1.485 (2)
Sr1—O11	2.5448 (13)	B2—O7	1.469 (2)
Sr1—O12	2.5255 (15)	B2—O8	1.479 (2)
Sr2—Cu	3.4028 (3)	O1—H1	0.82 (3)
Sr2—Cu^iv^	3.7257 (3)	O2—H2	0.77 (3)
Sr2—B2	3.230 (2)	O3—H3	0.82 (2)
Sr2—B2^v^	3.250 (2)	O4—H4	0.89 (3)
Sr2—O5^vi^	2.6250 (13)	O5—H5	0.82 (3)
Sr2—O6^v^	2.7961 (13)	O6—H6	0.79 (3)
Sr2—O6	2.5595 (12)	O7—H7	0.78 (2)
Sr2—O7^v^	2.5914 (13)	O8—H8	0.73 (3)
Sr2—O8	2.6730 (13)	O9—H9	0.78 (2)
Sr2—O9	2.5620 (13)	O10—H10	0.75 (3)
Sr2—O9^iv^	2.5267 (13)	O11—H11	0.82 (2)
Sr2—O10	2.5296 (14)	O12—H12	0.72 (3)
Sr2—H10	2.83 (3)		
			
Cu—Sr1—Cu^i^	111.901 (5)	O9^iv^—Sr2—O7^v^	151.18 (4)
B1^ii^—Sr1—Cu^i^	140.29 (4)	O9—Sr2—O7^v^	75.86 (4)
B1—Sr1—Cu^i^	90.00 (4)	O9^iv^—Sr2—O8	76.98 (4)
B1—Sr1—Cu	136.70 (4)	O9—Sr2—O8	150.04 (4)
B1^ii^—Sr1—Cu	89.41 (4)	O9^iv^—Sr2—O9	75.64 (5)
B1—Sr1—B1^ii^	96.70 (5)	O9^iv^—Sr2—O10	85.48 (4)
O1^iii^—Sr1—Cu^i^	55.25 (3)	O9^iv^—Sr2—H10	98.9 (6)
O1^iii^—Sr1—Cu	112.83 (3)	O9—Sr2—H10	74.6 (5)
O1^iii^—Sr1—B1	110.35 (5)	O10—Sr2—Cu^iv^	113.76 (3)
O1^iii^—Sr1—B1^ii^	86.00 (4)	O10—Sr2—Cu	34.87 (3)
O1^iii^—Sr1—O2^ii^	74.11 (4)	O10—Sr2—B2^v^	100.51 (5)
O1^iii^—Sr1—O3^ii^	86.24 (4)	O10—Sr2—B2	102.07 (5)
O1^iii^—Sr1—O4	85.65 (4)	O10—Sr2—O5^vi^	143.85 (4)
O2—Sr1—Cu	114.33 (3)	O10—Sr2—O6^v^	123.60 (4)
O2^ii^—Sr1—Cu	116.43 (3)	O10—Sr2—O6	83.73 (4)
O2^ii^—Sr1—Cu^i^	119.92 (3)	O10—Sr2—O7^v^	86.32 (4)
O2—Sr1—Cu^i^	115.87 (3)	O10—Sr2—O8	124.66 (4)
O2—Sr1—B1	27.04 (5)	O10—Sr2—O9	64.78 (4)
O2^ii^—Sr1—B1^ii^	27.13 (4)	O10—Sr2—H10	14.8 (5)
O2^ii^—Sr1—B1	78.43 (4)	Sr1—Cu—Sr1^i^	68.098 (5)
O2—Sr1—B1^ii^	81.29 (5)	Sr1—Cu—Sr2^iv^	112.094 (6)
O2—Sr1—O1^iii^	130.80 (4)	Sr2—Cu—Sr1^i^	111.304 (6)
O2—Sr1—O2^ii^	73.23 (5)	Sr2—Cu—Sr1	179.289 (7)
O2—Sr1—O3^ii^	100.66 (4)	Sr2^iv^—Cu—Sr1^i^	177.080 (7)
O2—Sr1—O4	53.81 (4)	Sr2—Cu—Sr2^iv^	68.482 (6)
O2—Sr1—O11	147.73 (4)	O9—Cu—Sr1	132.38 (4)
O3^ii^—Sr1—Cu^i^	138.39 (3)	O9—Cu—Sr1^i^	143.20 (4)
O3^ii^—Sr1—Cu	65.76 (3)	O9—Cu—Sr2^iv^	38.85 (4)
O3^ii^—Sr1—B1	120.90 (4)	O9—Cu—Sr2	48.33 (4)
O3^ii^—Sr1—B1^ii^	25.91 (4)	O9—Cu—O10	88.64 (6)
O3^ii^—Sr1—O2^ii^	51.33 (4)	O9—Cu—O12	92.85 (6)
O3^ii^—Sr1—O4	132.21 (4)	O10—Cu—Sr1^i^	94.45 (4)
O4—Sr1—Cu^i^	66.17 (3)	O10—Cu—Sr1	132.02 (4)
O4—Sr1—Cu	156.76 (3)	O10—Cu—Sr2	47.45 (4)
O4—Sr1—B1^ii^	106.49 (5)	O10—Cu—Sr2^iv^	83.30 (4)
O4—Sr1—B1	27.04 (4)	O11—Cu—Sr1	47.21 (4)
O4—Sr1—O2^ii^	81.21 (4)	O11—Cu—Sr1^i^	37.52 (4)
O11—Sr1—Cu^i^	84.18 (3)	O11—Cu—Sr2	132.08 (4)
O11^i^—Sr1—Cu^i^	27.81 (3)	O11—Cu—Sr2^iv^	140.44 (4)
O11—Sr1—Cu	33.96 (3)	O11—Cu—O9	179.26 (4)
O11^i^—Sr1—Cu	92.07 (3)	O11—Cu—O10	91.44 (6)
O11^i^—Sr1—B1^ii^	165.57 (5)	O11—Cu—O12	87.07 (6)
O11^i^—Sr1—B1	92.05 (5)	O12—Cu—Sr1	46.70 (4)
O11—Sr1—B1^ii^	99.45 (5)	O12—Cu—Sr1^i^	84.30 (4)
O11—Sr1—B1	160.81 (5)	O12—Cu—Sr2	133.81 (4)
O11—Sr1—O1^iii^	81.18 (4)	O12—Cu—Sr2^iv^	97.93 (4)
O11^i^—Sr1—O1^iii^	80.19 (4)	O12—Cu—O10	178.50 (5)
O11^i^—Sr1—O2^ii^	147.20 (4)	Sr1—B1—Sr1^ii^	83.30 (5)
O11—Sr1—O2^ii^	120.24 (4)	O1—B1—Sr1^ii^	144.15 (11)
O11^i^—Sr1—O2	111.00 (4)	O1—B1—Sr1	119.69 (11)
O11—Sr1—O3^ii^	73.88 (4)	O1—B1—O2	112.02 (15)
O11^i^—Sr1—O3^ii^	147.00 (4)	O2—B1—Sr1	50.65 (8)
O11—Sr1—O4	149.93 (4)	O2—B1—Sr1^ii^	59.44 (8)
O11^i^—Sr1—O4	76.79 (4)	O3—B1—Sr1	130.66 (11)
O11^i^—Sr1—O11	74.40 (5)	O3—B1—Sr1^ii^	50.85 (8)
O12—Sr1—Cu^i^	115.42 (3)	O3—B1—O1	109.14 (13)
O12—Sr1—Cu	34.22 (3)	O3—B1—O2	105.54 (14)
O12—Sr1—B1^ii^	101.14 (5)	O3—B1—O4	113.91 (15)
O12—Sr1—B1	102.96 (5)	O4—B1—Sr1	55.55 (8)
O12—Sr1—O1^iii^	144.89 (4)	O4—B1—Sr1^ii^	105.08 (11)
O12—Sr1—O2^ii^	124.65 (4)	O4—B1—O1	110.63 (14)
O12—Sr1—O2	84.31 (4)	O4—B1—O2	105.51 (14)
O12—Sr1—O3^ii^	86.10 (4)	Sr2—B2—Sr2^v^	83.90 (5)
O12—Sr1—O4	123.75 (4)	O5—B2—Sr2	118.97 (11)
O12—Sr1—O11^i^	87.98 (5)	O5—B2—Sr2^v^	145.53 (12)
O12—Sr1—O11	63.78 (4)	O5—B2—O6	113.74 (15)
Cu—Sr2—Cu^iv^	111.519 (5)	O5—B2—O8	112.96 (15)
Cu^iv^—Sr2—H10	127.6 (6)	O6—B2—Sr2^v^	59.15 (8)
Cu—Sr2—H10	41.1 (5)	O6—B2—Sr2	50.63 (8)
B2—Sr2—Cu^iv^	87.47 (4)	O7—B2—Sr2	131.87 (11)
B2^v^—Sr2—Cu	90.14 (4)	O7—B2—Sr2^v^	51.02 (8)
B2^v^—Sr2—Cu^iv^	144.03 (4)	O7—B2—O5	108.75 (14)
B2—Sr2—Cu	136.64 (4)	O7—B2—O6	105.14 (14)
B2—Sr2—B2^v^	96.10 (5)	O7—B2—O8	111.98 (15)
B2—Sr2—H10	96.2 (5)	O8—B2—Sr2	55.04 (8)
B2^v^—Sr2—H10	87.7 (6)	O8—B2—Sr2^v^	101.23 (10)
O5^vi^—Sr2—Cu^iv^	55.81 (3)	O8—B2—O6	104.01 (14)
O5^vi^—Sr2—Cu	111.74 (3)	Sr1^vi^—O1—H1	98 (2)
O5^vi^—Sr2—B2	111.12 (5)	B1—O1—Sr1^vi^	126.32 (10)
O5^vi^—Sr2—B2^v^	90.05 (4)	B1—O1—H1	113 (2)
O5^vi^—Sr2—O6^v^	78.53 (4)	Sr1—O2—Sr1^ii^	106.77 (5)
O5^vi^—Sr2—O8	85.09 (4)	Sr1—O2—H2	130 (2)
O5^vi^—Sr2—H10	152.7 (5)	Sr1^ii^—O2—H2	109 (2)
O6—Sr2—Cu^iv^	112.65 (3)	B1—O2—Sr1^ii^	93.44 (10)
O6^v^—Sr2—Cu	117.19 (3)	B1—O2—Sr1	102.31 (10)
O6^v^—Sr2—Cu^iv^	122.49 (3)	B1—O2—H2	109 (2)
O6—Sr2—Cu	114.91 (3)	Sr1^ii^—O3—H3	129.2 (18)
O6^v^—Sr2—B2^v^	27.13 (4)	B1—O3—Sr1^ii^	103.24 (10)
O6—Sr2—B2^v^	80.59 (5)	B1—O3—H3	115.3 (18)
O6^v^—Sr2—B2	77.68 (4)	Sr1—O4—H4	118.4 (13)
O6—Sr2—B2	26.66 (5)	B1—O4—Sr1	97.41 (10)
O6—Sr2—O5^vi^	132.31 (4)	B1—O4—H4	111.2 (13)
O6—Sr2—O6^v^	72.12 (4)	Sr2^iii^—O5—H5	101 (2)
O6—Sr2—O7^v^	100.74 (4)	B2—O5—Sr2^iii^	121.20 (11)
O6—Sr2—O8	52.97 (4)	B2—O5—H5	116 (2)
O6—Sr2—O9	148.41 (4)	Sr2—O6—Sr2^v^	107.88 (4)
O6^v^—Sr2—H10	109.2 (6)	Sr2^v^—O6—H6	104 (2)
O6—Sr2—H10	74.1 (5)	Sr2—O6—H6	130 (2)
O7^v^—Sr2—Cu	66.56 (3)	B2—O6—Sr2^v^	93.72 (10)
O7^v^—Sr2—Cu^iv^	142.15 (3)	B2—O6—Sr2	102.71 (10)
O7^v^—Sr2—B2^v^	26.14 (4)	B2—O6—H6	112 (2)
O7^v^—Sr2—B2	120.76 (4)	Sr2^v^—O7—H7	128.5 (18)
O7^v^—Sr2—O5^vi^	88.70 (4)	B2—O7—Sr2^v^	102.84 (10)
O7^v^—Sr2—O6^v^	51.45 (4)	B2—O7—H7	114.7 (18)
O7^v^—Sr2—O8	129.48 (4)	Sr2—O8—H8	110 (2)
O7^v^—Sr2—H10	77.5 (5)	B2—O8—Sr2	98.00 (10)
O8—Sr2—Cu	158.47 (3)	B2—O8—H8	115.9 (19)
O8—Sr2—Cu^iv^	66.01 (3)	Sr2^iv^—O9—Sr2	104.36 (5)
O8—Sr2—B2	26.96 (4)	Sr2—O9—H9	118 (2)
O8—Sr2—B2^v^	103.66 (5)	Sr2^iv^—O9—H9	111.7 (19)
O8—Sr2—O6^v^	78.24 (4)	Cu—O9—Sr2	97.21 (5)
O8—Sr2—H10	121.9 (5)	Cu—O9—Sr2^iv^	112.35 (6)
O9^iv^—Sr2—Cu^iv^	28.81 (3)	Cu—O9—H9	112 (2)
O9^iv^—Sr2—Cu	91.73 (3)	Sr2—O10—H10	106 (2)
O9—Sr2—Cu	34.46 (3)	Cu—O10—Sr2	97.68 (5)
O9—Sr2—Cu^iv^	84.10 (3)	Cu—O10—H10	102.7 (19)
O9^iv^—Sr2—B2	87.99 (5)	Sr1^i^—O11—Sr1	105.60 (5)
O9—Sr2—B2^v^	101.76 (5)	Sr1—O11—H11	119.6 (19)
O9—Sr2—B2	159.41 (5)	Sr1^i^—O11—H11	107.9 (19)
O9^iv^—Sr2—B2^v^	171.84 (5)	Cu—O11—Sr1^i^	114.67 (6)
O9—Sr2—O5^vi^	79.28 (4)	Cu—O11—Sr1	98.84 (5)
O9^iv^—Sr2—O5^vi^	81.88 (4)	Cu—O11—H11	110.3 (19)
O9^iv^—Sr2—O6	105.75 (4)	Sr1—O12—H12	147 (2)
O9—Sr2—O6^v^	122.57 (4)	Cu—O12—Sr1	99.08 (6)
O9^iv^—Sr2—O6^v^	149.49 (4)	Cu—O12—H12	112 (2)
			
Sr1—B1—O1—Sr1^vi^	94.45 (11)	O3—B1—O2—Sr1^ii^	−22.29 (12)
Sr1^ii^—B1—O1—Sr1^vi^	−29.6 (2)	O3—B1—O4—Sr1	124.07 (13)
Sr1—B1—O2—Sr1^ii^	108.00 (6)	O4—B1—O1—Sr1^vi^	155.69 (11)
Sr1^ii^—B1—O2—Sr1	−108.00 (6)	O4—B1—O2—Sr1^ii^	98.62 (12)
Sr1—B1—O3—Sr1^ii^	−26.13 (15)	O4—B1—O2—Sr1	−9.38 (14)
Sr1^ii^—B1—O4—Sr1	70.64 (8)	O4—B1—O3—Sr1^ii^	−90.35 (14)
Sr2^v^—B2—O5—Sr2^iii^	27.9 (3)	O5—B2—O6—Sr2	−108.88 (13)
Sr2—B2—O5—Sr2^iii^	−98.41 (11)	O5—B2—O6—Sr2^v^	141.85 (13)
Sr2^v^—B2—O6—Sr2	109.27 (6)	O5—B2—O7—Sr2^v^	−147.70 (11)
Sr2—B2—O6—Sr2^v^	−109.27 (6)	O5—B2—O8—Sr2	110.23 (13)
Sr2—B2—O7—Sr2^v^	24.67 (16)	O6—B2—O5—Sr2^iii^	−41.68 (19)
Sr2^v^—B2—O8—Sr2	−74.28 (7)	O6—B2—O7—Sr2^v^	−25.54 (14)
O1—B1—O2—Sr1^ii^	−140.94 (12)	O6—B2—O8—Sr2	−13.57 (13)
O1—B1—O2—Sr1	111.06 (12)	O7—B2—O5—Sr2^iii^	75.10 (16)
O1—B1—O3—Sr1^ii^	145.47 (11)	O7—B2—O6—Sr2	132.25 (11)
O1—B1—O4—Sr1	−112.56 (12)	O7—B2—O6—Sr2^v^	22.98 (12)
O2—B1—O1—Sr1^vi^	38.27 (18)	O7—B2—O8—Sr2	−126.59 (13)
O2—B1—O3—Sr1^ii^	24.91 (14)	O8—B2—O5—Sr2^iii^	−159.94 (11)
O2—B1—O4—Sr1	8.79 (13)	O8—B2—O6—Sr2^v^	−94.86 (12)
O3—B1—O1—Sr1^vi^	−78.22 (16)	O8—B2—O6—Sr2	14.41 (14)
O3—B1—O2—Sr1	−130.29 (11)	O8—B2—O7—Sr2^v^	86.77 (14)

Symmetry codes: (i) −*x*+1, −*y*+1, −*z*; (ii) −*x*, −*y*+2, −*z*; (iii) *x*+1, *y*, *z*; (iv) −*x*+1, −*y*+1, −*z*+1; (v) −*x*+2, −*y*, −*z*+1; (vi) *x*−1, *y*, *z*.

### Strontium copper(II) tetrahydroxide bis(tetrahydroxyborate) Hydrogen-bond geometry (Å, º)

**Table:**

*D*—H···*A*	*D*—H	H···*A*	*D*···*A*	*D*—H···*A*
O1—H1···O12^vii^	0.81 (3)	2.10 (3)	2.886 (2)	164 (3)
O2—H2···O3^viii^	0.77 (3)	2.07 (3)	2.8060 (18)	162 (3)
O3—H3···O5^ix^	0.82 (2)	2.07 (2)	2.8563 (19)	163 (2)
O4—H4···O8^x^	0.89 (2)	1.93 (2)	2.8125 (18)	176 (2)
O5—H5···O10^xi^	0.81 (3)	2.01 (3)	2.7961 (19)	163 (3)
O6—H6···O7^xii^	0.79 (3)	2.05 (3)	2.8129 (18)	162 (3)
O7—H7···O1^xiii^	0.78 (2)	2.10 (2)	2.8592 (19)	166 (2)
O8—H8···O12^iv^	0.74 (3)	2.35 (3)	3.073 (2)	166 (3)
O12—H12···O10^vi^	0.72 (3)	2.39 (3)	3.1076 (19)	171 (3)

Symmetry codes: (iv) −*x*+1, −*y*+1, −*z*+1; (vi) *x*−1, *y*, *z*; (vii) −*x*, −*y*+1, −*z*; (viii) −*x*−1, −*y*+2, −*z*; (ix) *x*−2, *y*+1, *z*−1; (x) *x*−1, *y*+1, *z*−1; (xi) −*x*+2, −*y*+1, −*z*+1; (xii) −*x*+3, −*y*, −*z*+1; (xiii) *x*+2, *y*−1, *z*+1.
